# Diet and mortality rates in Sub-Saharan Africa: Stages in the nutrition transition

**DOI:** 10.1186/1471-2458-11-801

**Published:** 2011-10-13

**Authors:** Zulfa Abrahams, Zandile Mchiza, Nelia P Steyn

**Affiliations:** 1 Centre for the Study of Social and Environmental Determinants of Nutrition, Human Sciences Research Council (HSRC), Cape Town, South Africa

## Abstract

**Background:**

During the last century we have seen wide-reaching changes in diet, nutritional status and life expectancy. The change in diet and physical activity patterns has become known as the nutrition transition. At any given time, a country or region within a country may be at different stages within this transition. This paper examines a range of nutrition-related indicators for countries in Sub-Saharan Africa (SSA) and attempts to develop a typical model of a country in transition.

**Methods:**

Based on the availability of data, 40 countries in SSA were selected for analysis. Data were obtained from the World Health Organisation, Demographic and Health Surveys and the Food and Agriculture Organisation of the United Nations. Multiple linear regression analysis (MLRA) was used to explore the determinants of infant mortality. A six point score was developed to identify each country's stage in the nutrition transition.

**Results:**

MLRA showed that underweight-for-age, protein and the percentage of exclusively breastfed infants were associated with the infant mortality rate (IMR). The majority of countries (n = 26) used in the analysis had nutrition transition scores of zero and one. Most of them had a high prevalence of infant mortality, children that were stunted or underweight-for-age, small percentages of women that were overweight and obese, and low intakes of energy, protein, and fat. Countries with the highest scores include South Africa, Ghana, Gabon, Cape Verde and Senegal which had relatively low IMRs, high levels of obesity/overweight, and low levels of underweight in women, as well as high intakes of energy and fat. These countries display classic signs of a population well established in the nutrition-related non-communicable disease phase of the nutrition transition.

**Conclusions:**

Countries in SSA are clearly undergoing a nutrition transition. More than half of them are still in the early stage, while a few have reached a point where changes in dietary patterns are affecting health outcomes in a large portion of the population. Those in the early stage of the transition are especially important, since primordial prevention can still be introduced.

## Background

Developed (high-income) and developing (low-to-medium income) countries differ significantly in causes of death. The leading causes of mortality in developed countries are non-communicable diseases (NCD) such as type 2 diabetes, cardiovascular diseases and cancer which account for approximately two-thirds of all deaths [1]. However, these diseases account for only one-third of deaths in the developing world, where infectious diseases and malnutrition, particularly in infants, account for up to 43 percent of deaths [1]. Furthermore, NCDs that used to be associated with old age in high-income countries are becoming more prevalent in the developing world following the rapid increase in industrialization, urbanization, economic development and globalization [1].

During the last century we have seen wide-reaching changes in diet, nutritional status, disease patterns and life expectancy. These changes are thought to occur as three separate transition processes. The demographic transition refers to a change from a period of high fertility and mortality to one of low fertility and mortality which occurs as a result of an increase in income, education and employment [2], while the epidemiological transition refers to a change from a period of high prevalence of infectious disease associated with poor sanitation, famine and malnutrition, to a period of high prevalence of chronic and degenerative diseases [3,4].

A period of transition thought to precede or occur simultaneously with the demographic and epidemiological transitions is the nutrition transition, resulting from a change in diet and physical activity patterns [5]. Popkin refers to this transition occurring in five stages.

During the first stage (hunter-gatherer), diets were high in carbohydrates and fibre and low in fat (especially saturated fat). Activity levels were high, and obesity levels low. The second stage (famine) refers to a period where food was scarce and dietary diversity low. While the type of physical activity done may have changed, the activity levels remained much the same. These changes are related to a shift toward settlements and cultivation, first of crops and later also livestock and poultry. During the third stage (receding famine), the amount of carbohydrates in the diet decreased, and fruit, vegetables and protein consumption increased. Physical activity levels started to decrease. This is the development of more technically advanced and productive agriculture. The fourth stage (nutrition-related non-communicable disease) is characterized by a diet high in fat, refined carbohydrates, sugar and cholesterol and low in fibre. Physical activity levels are low, and obesity prevalence high. The final stage (behavioural change) occurs due to a desire to prolong health and delay or prevent degenerative diseases. The consumption of complex carbohydrates, fruit and vegetables increases, while the consumption of fat, processed foods, meat and dairy products are reduced [4-6]. At any given time, a country or region within a country may be at a different stage within this transition.

While most high-income, developed countries seem to be in the final stage of the nutrition transition [5]; developing countries [6] have not been immune to this transition. Poor countries are plagued by the same risk factors (diets high in sugar, fat and alcohol, obesity, tobacco smoking and lack of physical activity) as their rich counterparts [7]. Furthermore, rural and urban areas within the same country may be at different stages of the transition.

Urban residents have access to a wider range of food products, many of which are high in fat and sugar. In addition, an increasing number of women are working, leaving less time for growing and producing food, shopping for ingredients, and preparing the often energy-intensive staples of traditional diets. Families therefore rely on processed foods, resulting in the development of obesity [1]. This is further exacerbated by the sedentary activities associated with urban jobs and the increase in the availability of televisions and computers.

Sub-Saharan Africa (SSA), made up of low- and middle-income countries, is undergoing a health transition causing these countries to experience a double burden of disease. The nutrition transition appears to be accelerating in SSA from the receding famine stage to the nutrition-related non-communicable disease stage. As a result, an increasing number of households are experiencing the dual burden of underweight and obesity occurring simultaneously [8,9]. At the same time high rates of NCDs, like diabetes, cardiovascular disease and cancer are occurring together with infectious diseases such as HIV/AIDS, tuberculosis and malaria [10].

Community studies from South Africa [11,12] have found that underweight and stunting coexist with overweight and obesity. In a study by Garrett and Ruel [13], using demographic and health survey (DHS) data from several developing countries, the relationship between stunted children and overweight and obese mothers was confirmed. In SSA, the percentage of stunted children with overweight mothers ranged from 0.6% in Mozambique to 8.2% in Namibia.

Countries in SSA are currently experiencing changes associated with the advancing nutrition transition, while at the same time struggling to eradicate the high prevalence of infant and child mortality prevalent in developing countries. This paper examines the role of a number of indicators associated with the advancing nutrition transition, as well as the prevalence of infant mortality and the factors impacting on its prevalence in SSA.

In this paper we hypothesise that countries in the advanced stage of the transition have adopted 'western' diets and are now plagued by high levels of overweight and obesity, and high levels of NCD mortality. They generally have fewer people living in poverty. Moreover, their levels of underweight-for-age, stunting and IMR may have decreased. Based on this hypothesis, a scoring system was developed and used to define a typical model of a country in transition.

## Methods

The current study is a secondary analysis of existing data available on IMR and mortality due to chronic NCD (NCD MR) in SSA, as well as their determinants. Forty countries in SSA were selected based on the availability of data from primary sources World Health Organisation (WHO) [14], Demographic and Health Surveys (DHS) [15] and the Food and Agriculture Organisation of the United Nations (FAO) [16]. Nutrition-related indicators which may have influenced the nutrition transition, as well as indicators associated with infant and child mortality were selected from the data available. A list of the key indicators and their definitions is shown in Table 1.

**Table 1:** Definition of key indicators

Indicators	Definition
Infant Mortality Rate (IMR)	Probability of dying between birth and age 1 per 1 000 live births
Stunting	Children < 5 years that are stunted for age (%) [Height-for-age Z-score ≤ -2SD]
Underweight-for-age	Children < 5 years that are underweight for age (%) [Underweight-for-age Z-score ≤ -2SD]
Low Birth Weight	Low-birth-weight newborns (%)
Exclusively Breastfed	Infants exclusively breastfed for the first 6 months of life (%)
Non-communicable Disease Mortality Rate (NCD MR)	Age-standardized mortality rate by cause (per 100 000 population)-due to NCD
Underweight	Adult women with a BMI < 18.5 (%)
Normal Weight	Adult women with a BMI of 18.5-24.9 (%)
Overweight/obese	Adult women with a BMI > 25 (%)
PPP	Population living on < $1 a day (%)
HIV prevalence	Prevalence of HIV among adults aged 15-49 (%)
Dietary energy supply (DES)	Dietary energy supply (kcal/capita/day)
Protein	Protein supply quantity (g/capita/day)
Energy from protein	Energy obtained from protein (%)
Fat	Fat supply quantity (g/capita/day)
Energy from fat	Energy obtained from fat (%)
Carbohydrates	Carbohydrate supply quantity (g/capita/day)
Energy from carbohydrates	Energy obtained from carbohydrates (%)

The most recent data (2000-2008) from the WHO Global Health Observatory Database [14] were used to obtain information on each country's IMR, the percentage stunting, underweight-for-age, low birth weight and exclusively breastfed infants, NCD mortality, prevalence of HIV among adults, and the percentage of the population living on less than one dollar per day (PPP). Zere and McIntyre [17] provided information on the prevalence of stunting and underweight in South African children younger than five years.

Country reports from the most recent DHS [15] were used to obtain information on mean Body Mass Index (BMI) of adult women. BMI was stratified into the percentage of women who were underweight (BMI < 18.5), were within the normal weight range (BMI = 18.5-24.9), or were overweight and obese (BMI > 25).

The 2008 food balance sheets from FAO [16] were used to gather information on dietary energy supply (DES), protein and fat per capita available in each country. The information obtained was used to generate values for carbohydrate supply. In addition, values for the percentage of energy from protein [(grams of protein ×4 calories) ÷ (calories of energy ×100)], fat [(grams of fat ×9 calories) ÷ (calories of energy ×100)] and carbohydrates [(grams of carbohydrates ×4 calories) ÷ (calories of energy ×100)] were calculated.

The IMR was selected as an independent variable. Pearson and Spearman correlations were then used to identify associations between IMR and various predictor variables. Multiple linear regression analysis was used to explore the determinants of IMR using possible predictor variables. All variables that may have an impact on IMR were included in the analysis, irrespective of results obtained from Pearson and Spearman correlations. The predictor variables used were stunting, underweight-for-age, PPP, HIV prevalence, energy, protein, fat, carbohydrates, energy from protein, energy from fat and energy from carbohydrates.

A scoring system was developed to help identify the stage of nutrition transition in each country. Indicators included in the scoring system were based on trends associated with the advancing nutrition transition. The advanced stage of the transition is associated with diets high in energy and fat, growing levels of overweight/obesity, decreasing levels of infant mortality and stunting, high levels of NCD mortality [7], and possibly a smaller percentage of the population living on less than $1 per day.

A score of one was allocated to each country whose score for the following indicators were in the top quartile of the range: energy, percentage of energy from fat and overweight/obesity. Countries whose score for the following indicators were in the bottom quartile of the range were also allocated a score of one: PPP, IMR and stunting. All indicators that did not receive a score of one automatically received a score of zero. The highest possible score was six and the lowest was zero. A score of six is representative of a country in the last phase of the nutrition transition while zero indicates a country in very early stages of transition. A score close to zero would encapsulate the famine stage and would typically be a country with a low per capita energy intake, high IMR, high prevalence of stunting and low prevalence of obesity. A score close to six, on the other hand, would reflect a high energy intake, low IMR and stunting and a high prevalence of obesity.

## Results

From a total of 48 countries in SSA, sufficient recent data were available for 40 countries. Those excluded were: Botswana, Djibouti, Equatorial Guinea, Mauritania, Mauritius, Seychelles, Somalia and Sudan. Table 1 defines the key indicators used. Tables 2 and 3 show the key indicators for 40 countries in SSA. A number of countries still have IMRs above 100 with the highest being Angola (130), Democratic Republic of Congo (DRC) (126), Chad (124) and Sierra Leone (123). Some countries still show a prevalence of stunting in more than 50% of children (Angola, Burundi, Ethiopia, Madagascar, Malawi, Niger and Rwanda). Another important nutrition indicator-exclusive breastfeeding was lower than 10% in some countries (Burkina Faso, Chad, Cote d'Ivoire, South Africa (RSA) and Gabon). In Angola, Niger and Sierra Leone the NCD MR was above 1000 deaths per 100 000. Countries in which more than three quarters of the population live on less than 1 dollar per day include Burundi, Liberia, Rwanda and Tanzania.

**Table 2:** Key Indicators from 40 countries in Sub-Saharan Africa

Country	Infant Mortality Rate	Stunting (%)	Underweight-for-Age (%)	Low birth weight (%)	Exclusively Breastfed (%)	NCD Mortality Rate	Under weight (%)	Normal Weight (%)	Overweight/Obese (%)
Angola*	130	50.8	27.5	12	11.1	1071	n/a	n/a	n/a
Benin	76	44.7	20.2	15	43.1	835	9.2	71.8	19
Burkina Faso	92	44.5	37.4	16	6.8	924	20.8	69.9	9.3
Burundi*	102	63.1	38.9	11	44.7	919	n/a	n/a	n/a
Cameroon	82	36.4	16.6	11	21.2	840	6.7	64.6	49.3
Cape Verde*	24	21.4	11.8	6	59.6	591	n/a	n/a	n/a
Central African Republic* (CAR)	115	44.6	21.8	13	23.1	868	n/a	n/a	n/a
Chad	124	44.8	33.9	22	2.1	910	20.3	72	9.6
Comoros	75	46.9	25.0	25	21.3	713	9.8	67.5	18.4
Congo Brazzaville*	80	31.2	11.8	13	19.1	716	n/a	n/a	n/a
Cote d'Ivoire*	81	40.1	16.7	17	4.3	946	n/a	n/a	n/a
Democratic Republic of Congo (DRC)	126	45.8	28.2	12	36.1	921	18.4	71.3	11.3
Eritrea*	41	43.7	34.5	14	52	686	37.3	53.8	8.9
Ethiopia	69	50.7	34.6	20	49	817	25.6	69.1	4.4
Gabon	57	26.3	8.8	14	5.2	716	6.6	64	29.5
Gambia*	80	27.6	15.8	20	40.8	830	n/a	n/a	n/a
Ghana	51	28.6	14.3	9	62.8	699	8.6	61.4	30
Guinea	90	40.0	20.8	12	48.1	844	13.2	72.5	14.3
Guinea-Bissau*	117	28.1	17.2	24	27.9	925	n/a	n/a	n/a
Kenya	81	35.8	16.5	10	n/a	729	12.3	62.6	25.1
Lesotho	63	45.2	16.6	13	36.4	581	5.7	52	42.3
Liberia	100	39.4	20.4	14	29.1	931	10	69.4	20.5
Madagascar	68	52.8	36.8	17	n/a	799	26.7	67	6.3
Malawi	65	53.2	15.5	13	56.7	796	9.2	77.1	13.6
Mali	102	38.5	27.9	19	37.8	967	13.5	68.9	17.6
Mozambique*	90	47	21.2	15	30	777	n/a	n/a	n/a
Namibia*	31	29.6	17.5	16	23.9	513	15.9	56	28.1
Niger	79	54.8	39.9	27	n/a	1030	19.2	67.9	22.7
Nigeria	96	41.0	26.7	14	13.1	909	12.2	65.7	22.1
Rwanda	72	51.7	18.0	6	88.4	878	9.8	78.7	11.5
Sao Tome and Principe*	64	35.2	10.1	8	n/a	788	7.7	58.7	33.7
Senegal*	57	20.1	14.5	19	34.1	852	n/a	n/a	n/a
Sierra Leone	123	37.4	21.3	24	11.2	1033	11.2	59.1	29.7
South Africa (RSA)	48	24.5^16^	17.0^16^	15	7.2	867	6.2	38.9	54.9
Swaziland	59	29.5	6.1	9	32.3	707	3.2	46.2	50.6
Tanzania	67	44.4	16.7	14	60.1	851	10.4	71.8	17.7
Togo	64	26.9	20.5	10	41.3	818	10.9	77.8	11.4
Uganda	84	38.7	16.4	12	48	786	12.1	71.3	16.5
Zambia	92	45.8	14.9	11	60.7	833	9.6	71.2	19.2
Zimbabwe*	62	35.8	14.0	11	22.2	816	9.2	65.8	25
Mean	79.5	39.7	21.1	14.8	33.6	826.8	13.1	65.5	22.4
Standard Deviation	25.5	10.0	8.7	5.1	20.2	121.8	7.3	9.1	13.2
Minimum-Maximum	24-130	20.1-63.1	6.1-39.9	6-27	2.1-88.4	513-1071	3.2-37.3	38.9-78.7	4.4-54.9

* Indicates countries with missing data

n/a indicates a missing data value

**Table 3:** Key nutrition Indicators and income status (poverty) from 40 countries in Sub-Saharan Africa

Country	PPP	HIV prevalence (%)	Energy (cal/capita/ day)	Protein (g/capita/day)	Energy from protein (%)	Fat (g/capita/day)	Energy from fat (%)	Carbohydrates (g/capita/day)	Energy from carbohydrates (%)
Angola	54.3	2.0	1973	44	8.92	47	21.44	343.5	69.64
Benin	47.3	1.2	2533	59.4	9.38	52.2	18.55	456.4	72.07
Burkina Faso	56.5	1.2	2677	81.4	12.16	58.6	19.70	456	68.14
Burundi	81.3	3.5	1685	45.6	10.82	13.4	7.16	345.5	82.02
Cameroon	32.8	5.3	2269	57.4	10.12	42.6	16.90	414	72.98
Cape Verde	20.6	n/a	2572	69.5	10.81	76.8	26.87	400.7	62.32
Central African Republic (CAR)	62.4	5.1	1986	46.1	9.28	64.9	29.41	304.4	61.31
Chad	61.9	3.4	2056	61.5	11.96	49.1	21.49	342	66.54
Comoros	46.1	0.1	1884	44.6	9.47	45.1	21.54	324.9	68.98
Congo Brazzaville	54.1	3.5	2512	52.7	8.39	60.7	21.75	438.7	69.86
Cote d'Ivoire	23.3	3.7	2528	49.9	7.90	47.2	16.80	513.3	81.22
Democratic Republic of Congo (DRC)	59.2	1.3	1605	24.4	6.08	24.3	13.63	322.2	80.30
Eritrea*	n/a	0.8	1605	47.3	11.79	25.4	14.24	296.8	73.97
Ethiopia	39	n/a	1980	56.7	11.45	21	9.55	391	78.99
Gabon	4.8	5.3	2755	81.3	11.80	58.5	19.11	475.8	69.08
Gambia	34.3	1.7	2385	55.7	9.34	68.4	25.81	386.6	64.84
Ghana	30	1.8	2907	59.8	8.23	51.3	15.88	551.5	75.89
Guinea	70.1	1.4	2568	54.4	8.47	62.9	22.04	446.1	69.49
Guinea-Bissau	48.8	2.5	2306	44.3	7.68	50.9	19.87	417.7	72.45
Kenya	19.7	6.3	2089	59.7	11.43	49.1	21.15	352.1	67.42
Lesotho	43.4	23.6	2476	69.4	11.21	32.6	11.85	476.25	76.94
Liberia	83.7	1.6	2204	36.5	6.62	57.1	23.32	386	70.05
Madagascar	67.8	0.2	2160	49.9	9.24	31.9	13.29	418.3	77.46
Malawi	73.9	11.2	2172	56.6	10.42	31.9	13.22	414.6	76.35
Mali	51.5	1.0	2614	71.7	10.97	54.7	18.83	458.7	70.19
Mozambique	74.7	11.4	2067	37.9	7.33	37.7	16.42	394	76.25
Namibia*	n/a	13.7	2383	67.9	11.40	55.7	21.04	402.5	67.56
Niger	65.9	0.8	2376	80	13.47	46.5	17.61	409.4	68.92
Nigeria	64.4	3.6	2741	63.5	9.27	66.5	21.84	472.1	68.89
Rwanda	76.6	2.9	2085	49.5	9.50	20.1	8.68	426.5	81.82
Sao Tome and Principe*	n/a	n/a	2684	60.5	9.02	74.9	25.12	442	65.87
Senegal	33.5	0.8	2348	58.7	10.00	64.6	24.76	382.9	65.23
Sierra Leone	53.4	1.6	2170	52.2	9.62	56	23.23	364.3	67.15
South Africa (RSA)	26.2	17.9	2999	80.9	10.79	81.9	24.58	484.6	64.63
Swaziland	62.9	25.9	2292	60.9	10.63	49.1	19.28	401.6	70.09
Tanzania	88.5	5.8	2032	49.1	9.67	33.3	14.75	384	75.59
Togo	38.7	3.2	2161	48.9	9.05	46.9	19.53	385.8	71.41
Uganda	51.5	6.4	2211	48.7	8.81	42.6	17.34	408.2	73.85
Zambia	64.3	13.6	1873	46.6	9.95	34.4	16.53	344.3	73.53
Zimbabwe*	n/a	15.1	2238	55.9	9.99	56.9	22.88	375.6	67.13
Mean	51.8	5.69	2279	56.0	9.8	48.6	18.9	405.3	71.4
Standard Deviation	20.0	6.49	334.3	12.6	1.6	16.1	5.0	56.7	5.3
Minimum	4.8	0.1	1605	24.4	6.08	13.4	7.16	296.8	61.31
Maximum	88.5	25.9	2999	81.4	13.47	81.9	29.41	551.5	82.02

* Indicates countries with missing data

n/a indicates a missing data value

**Figure 1:**
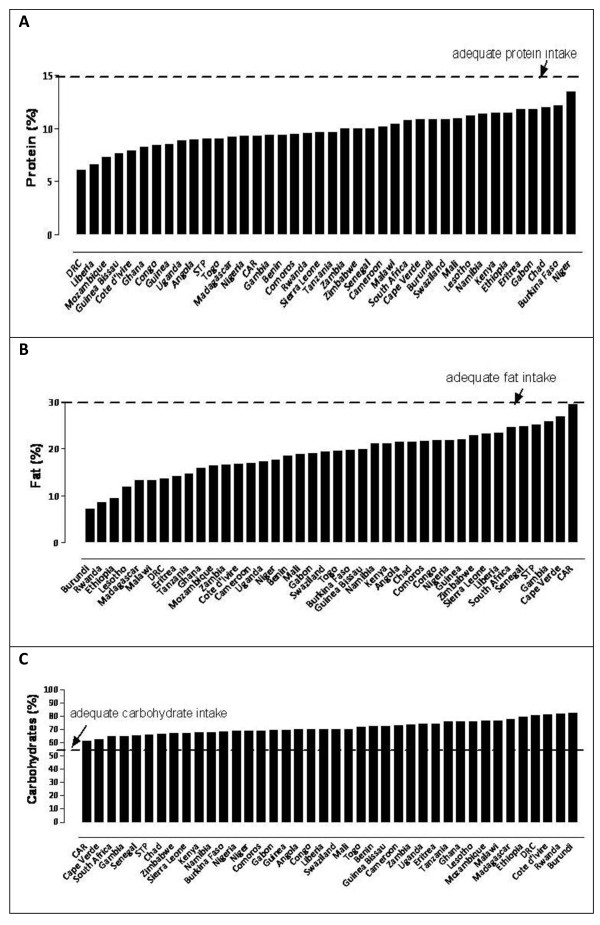
**Protein, fat and carbohydrate intake as a percentage of DES in SSA**. (A) protein as a percentage of DES, (B) fat as a percentage of DES, and (C) carbohydrates as a percentage of DES.

In terms of diet composition, results show that the average DES was 2279 ± 334 kcal/capita/day. Three countries had a DES less than 1800 (Burundi, DRC and Eritrea) and another 5 less than 2000 (Angola, Central African Republic (CAR), Comoros, Ethiopia and Zambia). Figures 1a, b and 1c show that the diet in SSA consists primarily of carbohydrates (> 55% of the overall DES) with protein supplying less than 15% of the overall DES. The average available protein intake was 56.0 ± 12.6 g/capita/day, supplying an average of 9.8 ± 1.6% of the overall DES. Countries with the lowest protein intakes (less than 45 g/capita/day) were Angola, Guinea-Bissau, Comoros, DRC, Liberia, and Mozambique. The average available fat intake was 48.6 ± 16 g/capita/day, which supplied an average of 18.9 ± 5% of the overall DES. Five countries had an extremely low fat intake of less than 30 g/capita/day: Burundi, DRC, Eritrea, Ethiopia, and Rwanda.

The results of correlations between independent variable IMR and several predictor variables are shown in Table 4. Correlations between IMR and predictor variables that were statistically significant include stunting (p = 0.01), underweight-for-age (p = 0.02), PPP (p = 0.02), energy (p = 0.04), protein (p = 0.01) and the percentage of energy from protein (0.04).

**Table 4:** Correlations between independent variables (infant mortality rate and non-communicable disease mortality rate) and several predictor variables

Independent Variable-Infant Mortality Rate	r**	P-Value
Stunting	0.41	0.01^†^
Underweight-for-Age*	0.49	0.02^†^
Low birth weight	0.31	0.06
Exclusive breastfeeding	-0.33	0.05
HIV prevalence*	-0.21	0.20
PPP	0.41	0.02^†^
Energy	-0.34	0.04^†^
Protein	-0.44	0.01^†^
Energy from protein	-0.35	0.04^†^
Fat	-0.14	0.53
Energy from fat	0.03	0.83
Carbohydrates	-0.31	0.08
Energy from carbohydrates	0.08	0.60

* Spearman's Rank Correlation (non-normal data)

** r is a measure of the linear relationship between IMR and the independent variables

^† ^Statistically significant at p < 0.05

Multiple logistic regression analysis was used to explore the determinants of IMR. All variables used in the univariate analysis (Table 3) were included, irrespective of whether they were significantly associated with the IMR. Table 5 shows that underweight-for-age (p = 0.02), protein (p = 0.001) and the percentage of exclusively breastfed infants (p = 0.003) are associated with the IMR. As the percentage of children that are underweight-for-age increases, the IMR increases. Conversely, an increase in the protein supply and number of exclusively breastfed infants results in a decrease in the IMR. Stunting, HIV prevalence, PPP, total energy, and energy from protein were no longer significant, whereas exclusive breastfeeding became significant. The determinants of NCD MR were explored and excluded since the NCD MR are estimates based on assumptions and extrapolations due to the lack of reliable mortality and morbidity data from SSA [18].

**Table 5:** Results of multiple linear regression analysis using the outcome variable infant mortality rate

Outcome: Infant Mortality Rate				
	**β Coefficient**	**Standard Error**	**P-value**	**95% confidence interval**
	
Underweight-for-age*	1.03	0.41	0.020	0.17-1.89
Protein**	-1.12	0.27	0.001	-1.67-(-0.56)
Exclusively Breastfed*	-0.55	-3.27	0.003	-0.90-(-0.21)
Constant	139.66	21.45	0.001	95.97-183.36

Interpretation				
				
Underweight-for-Age*	For every 1% increase in the number of children underweight-for-age, the infant mortality rate increases by 1 death per 1000 infants			
Protein^†^	For every 1 g increase in protein/capita/day, the infant mortality rate decreases by 1 death per 1000 infants			
Exclusively Breastfed*	For every 1% increase in the number of infants that are exclusively breastfed for 6 months, the infant mortality rate decreases by 0.5 deaths per 1000 infants			

* Indicates measurement in percentage

^† ^Indicates measurement in grams/capita/day

**Figure 2:**
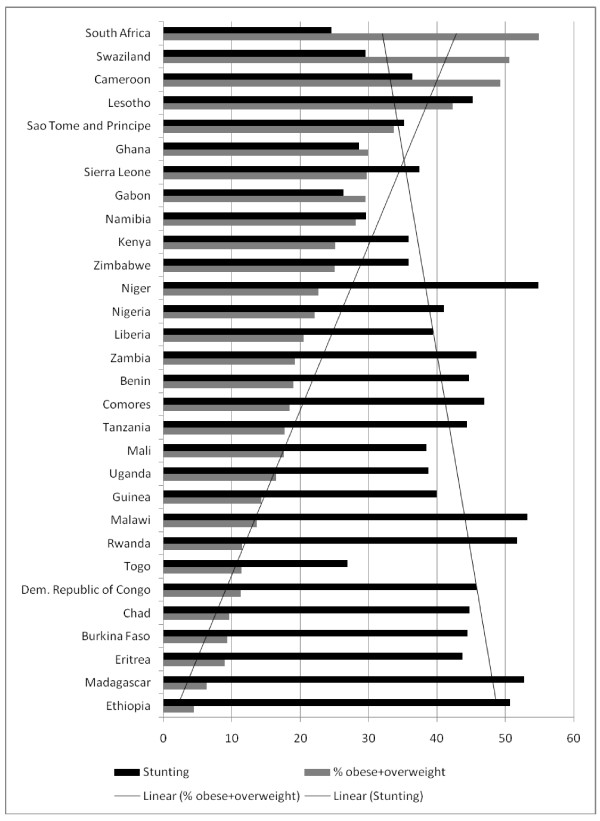
**Relationship between Stunted children and overweight/obese women**.

Figure 2 shows the relationship between stunted children and overweight/obese women. Findings show that, to differing degrees, all countries have stunted children and overweight/obese woman. The percentages of stunted children coupled with overweight/obese women were especially high in Lesotho, Swaziland, Cameroon and Sao Tome and Principe.

A scoring system was developed to assess each country's stage in the nutrition transition (Table 6). Countries with the highest scores include RSA which scored a six, Ghana, Gabon and Cape Verde which scored five, and Senegal which scored four. These countries had relatively low IMR (24-57 deaths per 1000 live births), relatively high levels of obesity/overweight (> 29%), and low levels of underweight (6-9%) in women, as well as high intakes of energy (DES > 2500 kcal/capita/day) and fat (> 50 g/capita/day) and average NCD MR (591-867 deaths per 100 000). Countries having a zero score include Angola, Burundi, Chad, Comoros, Congo Brazzaville, DRC, Ethiopia, Madagascar, Malawi, Mozambique, Niger, Rwanda, Tanzania, Uganda and Zambia. They are characterized by many in the population living on less than 1 dollar per day (39-81%), a low DES (1600-2500cal/capita/day), low energy intake from fat (7-21%), a low prevalence of obesity (4-22%), and a high prevalence of stunting (31-63%). Although they do not all lie in the highest quartile for IMR it still remains high being between 65 and 124 in these countries.

**Table 6:** 40 Sub-Saharan countries listed in descending order of Nutritional Transition Scores and the indicators that contribute to them

Country	PPP		Energy		% Energy from fat		Over weight/obesity		IMR		Stunting		Score
	**Low**	**High**	**High**	**Low**	**High**	**Low**	**High**	**Low**	**Low**	**High**	**Low**	**High**	
		
Angola				X			n/a	n/a		X		X	0
Burundi		X		X		X	n/a	n/a		X		X	0
Chad				X				X		X			0
Comoros				X								X	0
Congo Brazzaville							n/a	n/a					0
Dem. Republic of Congo				X		X		X		X			0
Ethiopia				X		X		X				X	0
Madagascar		X				X		X				X	0
Malawi		X				X						X	0
Mozambique		X					n/a	n/a				X	0
Niger		X										X	0
Rwanda		X				X		X				X	0
Tanzania		X		X		X							0
Uganda													0
Zambia				X									0
Benin			**✔**										1
Burkina Faso			**✔**					X					1
Central African Republic				X	**✔**		n/a	n/a		X			1
Cote d'Ivoire	**✔**						n/a	n/a					1
Eritrea	n/a	n/a		X		X		X	**✔**				1
Guinea-Bissau							n/a	n/a		X	**✔**		1
Kenya	**✔**												1
Liberia		X			**✔**					X			1
Mali			**✔**							X			1
Nigeria			**✔**							X			1
Togo								X			**✔**		1
Cameroon	**✔**						**✔**						2
Guinea		X	**✔**		**✔**								2
Lesotho						X	**✔**		**✔**				2
Namibia	n/a	n/a							**✔**		**✔**		2
Sierra Leone					**✔**		**✔**			X			2
Zimbabwe	n/a	n/a			**✔**				**✔**				2
Gambia	**✔**				**✔**		n/a	n/a			**✔**		3
Sao Tome and Principe	n/a	n/a	**✔**		**✔**		**✔**						3
Swaziland							**✔**		**✔**		**✔**		3
Senegal	**✔**				**✔**		n/a	n/a	**✔**		**✔**		4
Cape Verde	**✔**		**✔**		**✔**		n/a	n/a	**✔**		**✔**		5
Gabon	**✔**		**✔**				**✔**		**✔**		**✔**		5
Ghana	**✔**		**✔**			X	**✔**		**✔**		**✔**		5
South Africa	**✔**		**✔**		**✔**		**✔**		**✔**		**✔**		6

**High **refers to countries whose levels were in the top quartile

**Low **refers to countries whose levels were in the bottom quartile

**n/a **refers to countries with no available data

**✔ **refers to levels included in the score

**X **refers to levels opposite to those included in the score

## Discussion

It has long been accepted that the health status of a population is dependent on many inter-connected factors. Environmental, socio-economic and political influences are all associated with attaining health. This study has looked at the impact of nutrition (DES, protein, carbohydrate and fat intakes) on IMR and undernutrition in children and in overnutrition in adults

One of the most important findings of this SSA study is the significant relationship between IMR with exclusive breastfeeding and available protein intake shown in the multiple regression analysis. Stunting, DES and PPP were not significant predictors of IMR, despite them being significant in the univariate analysis. It appears that the strong relationship between IMR and these variables were by chance, and once confounding was controlled for, the significant associations disappeared.

Indeed, the regression analysis in the current study showed exclusive breastfeeding to be significantly associated with IMR, even though it was insignificant in the univariate analysis. It is disheartening to know that only a small prevalence (33.6 ± 20%) of mothers in SSA countries chose to breastfeed exclusively, despite the abundance of strategies directed at promoting exclusive breastfeeding in the continent. This prevalence is corroborated by those of WHO [19] that reported a finding of 30% in SSA. Breast milk is always the best, especially in a continent that grapples with high levels of food insecurity, poverty and HIV/AIDS infections. Breast milk provides just the right blend of proteins, fats, carbohydrates, minerals, and calories. Furthermore, it contains antibodies, which help protect the baby from infections and diseases [20-22]. As such, the results of the current study have important implications for policy-makers in Africa, as it emphasizes the importance of exclusive breastfeeding in the prevention of infant mortality.

The current study also highlights the fact that many countries in SSA have at least a reasonable available intake of protein (> 10% of total DES) and fat (> 20% of total DES) [20]. The amount of protein consumed seems to be an important predictor for IMR in the current analysis. Indeed, results outlined in Figure 1a suggest that those individuals residing in DRC, Liberia and Guinea-Bissau consume the lowest amount of energy from protein, while at the same time having the highest levels of infant mortality (Table 2). Moreover, according to Earl and Borra [20], populations consuming 24-44 g of protein may not be getting enough essential amino acids. Amino acids are indeed crucial in the growth of infants; issues that have been stressed in child health for decades. Clearly, health workers in SSA need to prioritise strategies directed at emphasizing the importance of protein in the infants' diets as well as the promotion of exclusive breastfeeding for at least 6 months if the IMR is to be reduced.

The mean intake of 18.9 ± 5% of the overall DES from fat in the current study poses concern. Thirteen to 24 g of fat is equivalent to at least 2.5 to 5 teaspoons of fat. These levels are very low when compared to the recommended daily allowance of at least 65 g for adults and children over 4 years [20,21]. As such, those individuals (especially children) consuming small amounts of fat may not be getting enough essential fatty acids to sustain their daily needs. It is therefore not surprising that countries at the lower extremes of total fat intake (Burundi with 13.4 g and DRC with 24.3 g) also presented with high IMR of 102 and 126, respectively.

The fourth Millennium Development Goal (MDG) is to reduce child mortality. One of the indicators used to measure progress is the IMR [23], which is a commonly used indicator of a population's economic and social development [24]. Despite developing countries experiencing a decline in infant mortality during the last century, the results of the current study clearly show that the prevalence of undernutrition and infant mortality in SSA is still unacceptably high. These results are corroborated by other studies [25-27]. Moreover, according to Chopra and Darnton-Hill [28], the proportion and number of undernourished children in SSA has increased during the last 10 years.

Findings from this study indicate that the IMR in SSA ranges from as low as 24 to as high as 130 deaths per 1000 live births, while the percentage of stunted children range from 20% to 63%. Countries with the highest nutrition transition scores were found to have relatively low levels of IMR, stunted children and underweight-for-age. Despite some countries having lower levels than others, the percentage of children that are stunted and underweight-for-age in SSA as a whole, is still disappointingly high. From these results we conclude that countries experiencing the nutrition-related NCD phase of the transition, while experiencing high levels of obesity, are also experiencing a decline in rates for infant mortality and children that are stunted and underweight-for-age.

To speed up the reduction of infant and child mortality in SSA, countries need to allocate money to improving mother and child health care services and train health workers to detect and treat malnutrition related conditions as early as possible. Furthermore, improving the nutritional status of pregnant women will improve fetal growth which may lead to better health implications for many children [2].

Findings of the current study also show that RSA, Ghana, Gabon and Cape Verde had the highest nutrition transition scores. These countries with their relatively low levels of IMR and underweight, and relatively high levels of obesity/overweight, energy and fat intakes display classic signs of a population well established in the nutrition-related NCD phase of the nutrition transition.

However, countries with a high prevalence of obesity/overweight among women (Swaziland, Cameroon, Lesotho, and Sao Tome and Principe) were also found to have a moderate prevalence of children that are stunted (29-45%), a low prevalence of children that are underweight-for-age (6-17%), and nutrition transition scores of two and three. These findings confirm the prevalence of the dual burden household [9]. While the dual burden household appears to be prevalent throughout SSA, its prevalence is especially high in countries undergoing the receding famine stage of the nutrition transition. This is further supported by their average energy and fat intakes.

Findings show that more than half of the countries (n = 26) evaluated in this analysis had nutrition transition scores of zero and one. Most of them had a high prevalence of children that are stunted or underweight-for-age, small percentages of women that are overweight and obese, and low intakes of energy, protein, and fat. In addition, their IMR are moderate to high, and in most of these countries more than 50% of the population live on less than one dollar per day. These countries are still in the receding famine stage of the nutrition transition. This implies that within the next few years they can be expected to move into stages 3-5 with their typical pattern.

Since stunting and undernutrition during childhood is associated with obesity during adulthood [29], the co-morbidities related to obesity are bound to increase in countries still in the early stages of the transition. This will result in their moderate NCD mortality levels rising dramatically. As such, endorsing strategies directed at reducing food insecurity in these countries will directly reduce the prevalence of stunting and at the same time indirectly reduce the prevalence of chronic non-communicable diseases.

The scoring system developed and used in the analysis of this paper has attempted to identify each country's stage in the nutrition transition. This system has the potential to be applied to other developing countries, thereby identifying their stage in the transition and being able to predict areas of concern that are most in need of assistance.

## Conclusion

Findings from this study show that countries in SSA are undergoing a nutrition transition. More than half of them are still in the early stages, while a few have reached a point where changes in dietary patterns are affecting health outcomes in a large portion of the population. Countries in the early stage of the transition are especially important, since the speed of transition is occurring more rapidly in lower- and middle-income countries. These countries are at risk of developing a high prevalence of obesity and NCD mortality more rapidly than has occurred in the West.

## Competing interests

The authors declare that they have no competing interests.

## Authors' contributions

ZA: data collation, analysis and wrote the manuscript, and approved the final manuscript. NS: conceptualised paper, reviewed manuscript. Read and approved the final manuscript. ZM: involved in writing and reviewing manuscript. Read and approved final manuscript.

## Pre-publication history

The pre-publication history for this paper can be accessed here:

http://www.biomedcentral.com/1471-2458/11/801/prepub

## References

[B1] World Health Organisation Nutrition in transition: globalization and its impact on nutrition patterns and diet-related diseases 2003 https://apps.who.int/nut/trans.htm

[B2] Amuna P Zotor F Epidemiological and nutrition transition in developing countries: impact on human health and development Proc Nutr Soc 2008 67 82 90 10.1017/S0029665108006058 18234135

[B3] Omran AR The epidemiologic transition: a theory of the epidemiology of population change Milbank Mem Fund 1971 49 509 38 10.2307/3349375 5155251

[B4] Popkin BM An overview of the nutrition transition and its health implications: the Bellagio meeting Public Health Nutr 2002 5 93 103 12027297 10.1079/phn2001280

[B5] Popkin BM Nutritional Patterns and Transitions Population Devel Rev 1993 19 138 57 10.2307/2938388

[B6] Popkin BM The nutrition transition in low income countries: An emerging crisis Nutr Rev 1994 52 285 98 7984344 10.1111/j.1753-4887.1994.tb01460.x

[B7] Unwin N Alberti G Chronic non-communicable diseases Ann Trop Med Parasitol 2006 100 455 64 10.1179/136485906X97453 16899148

[B8] Popkin BM Part II. What is unique about the experience in lower- and middle-income less-industrialised countries compared with the very-high-income countries? Public Health Nutr 2002 5 1A 205 1412027286

[B9] Doak CM Adair LS Bentley M Monteiro C Popkin BM The dual burden household and the nutrition transition paradox Int Journ Obes 2005 29 129 31 10.1038/sj.ijo.0802824 15505634

[B10] Maher D Smeeth L Sekajugo J Health transition in Africa: practical policy proposals for primary care Bull World Health Organ 2010 88 943 48 10.2471/BLT.10.077891 21124720 PMC2995191

[B11] Bourne LT Langenhoven ML Steyn K Jooste PL Laubscher JA Bourne DE Nutritional status of 3-6 year-old African children in the Cape Peninsula East Afr Med J 1994 71 695 702 7859652

[B12] Steyn K Bourne L Jooste P Fourie JM Rossouw K Lombard C Anthropometric profile of a black population of the Cape Peninsula in South Africa East Afr Med J 1998 75 35 40 9604533

[B13] Garrett J Ruel M Stunted child-overweight mother pairs: an emerging policy concern? 2003 http://pdf.usaid.gov/pdf_docs/PNACT013.pdf

[B14] World Health Organisation Global Health Observatory Database: Country Statistics 2011 http://apps.who.int/ghodata/?theme=country

[B15] Demographic and Health Surveys Country Reports 2011 http://www.measuredhs.com/countries/

[B16] Food and Agriculture Organisation of the United States FAOSTAT: Food Balance Sheets 2011 http://faostat.fao.org/site/368/default.aspx#ancor

[B17] Zere A McIntyre D Inequities in under-five child malnutrition in South Africa Int J Equity Health 2003 2 7 10.1186/1475-9276-2-7 14514357 PMC201028

[B18] Unwin N Setel P Rashid S Magusi F Mbanya J Kitange H Hayes L Edwards R Aspray T Alberti Noncommunicable diseases in sub-Saharan Africa: where do they feature in the health research agenda? Bull World Health Organ 2001 79 10 947 953 11693977 PMC2566676

[B19] World Health Organisation Infant and young child feeding-Model Chapter for textbooks for medical students and allied health professionals 2009 http://www.waba.org.my/pdf/Infant-n-Young-Feeding.pdf 23905206

[B20] Earl R Borra ST Mahan LK, Escott-Stump S Guidelines for dietary planning Krause's food, nutrition, & diet therapy 2000 WB Saunders Co22009544

[B21] Laquatra I Mahan LK, Escott-Stump S Nutrition weight management Krause's food, nutrition, & diet therapy 2000 WB Saunders Co22009544

[B22] UNICEF Press Centre. 15 years after Innocenti Declaration, breastfeeding saving six million lives annually 2006 http://www.unicef.org/media/media_30011.html

[B23] Millennium Project Goals, targets, indicators 2006 http://www.unmillenniumproject.org/goals/gti.htm

[B24] Masuy-Stroobant G Gourbin C Infant health and mortality indicators: their accuracy for monitoring the socio-economic development in the Europe of 1994 Eur J Popul 1995 11 1 63 84 10.1007/BF01264105 12158978

[B25] Harrtgen K Misselhorn M A multilevel approach to explain child mortality and undernutrition in South Asia and Sub-Saharan Africa 2006 http://econstor.eu/bitstream/10419/19847/1/Misselhorn.pdf

[B26] Adetunji J Bos ER Jamison DT, Feachem RG, Makgoba MW, et al Levels and Trends in mortality in Sub-Saharan Africa: An Overview Disease and Mortality in Sub-Saharan Africa 2006 2 Washington (DC), World Bank

[B27] Garenne M Gakusi E Health transitions in sub-Saharan Africa: overview of mortality trends in children under 5 years old (1950-2000) Bulletin of the World Health Organ 2005 84 6 470 78 10.2471/blt.05.029231 PMC2627376 16799731

[B28] Chopra M Darnton-Hill I Responding to the crisis in sub-Saharan Africa: the role of nutrition Public Health Nutr 2006 9 5 544 50 16923284 10.1079/phn2006948

[B29] Adair LS Prentice AM A critical evaluation of the fetal origins hypothesis and its implications for developing countries J Nutr 2004 134 191 193 14704317 10.1093/jn/134.1.191

